# Supplementary material for: Metabarcoding the Bacterial Assemblages Associated with Toxopneustes roseus in the Mexican Central Pacific
Source: Microorganisms. 2024 Jun 13;12(6):1195. doi: 10.3390/microorganisms12061195 (PMC11205562; doi:10.3390/microorganisms12061195)
Supplement: Supplementary file 1 [file microorganisms-12-01195-s001.zip › microorganisms-3056265-supplementary.pdf]

Metabarcoding the Bacterial Assemblages Associated with
Toxopneustes roseus in the Mexican Central Pacific

Joicye Hernández-Zulueta [1,2,†], Sharix Rubio-Bueno [3], María del Pilar Zamora-Tavares [4], Ofelia Vargas-Ponce [4], Alma Paola Rodríguez-Troncoso [5] and Fabián A. Rodríguez-Zaragoza [2,*,†]

[1] Departamento de Biología Celular y Molecular, Centro Universitario de Ciencias Biológicas y
Agropecuarias (CUCBA), Universidad de Guadalajara, Zapopan 45200, Jalisco, Mexico; joicye.hernandez@academicos.udg.mx

[2] Laboratorio de Ecología Molecular, Microbiología y Taxonomía (LEMITAX), Departamento de Ecología Aplicada, Centro Universitario de Ciencias Biológicas y Agropecuarias (CUCBA), Universidad de
Guadalajara, Zapopan 45200, Jalisco, Mexico

[3] Programa de Maestría en Ciencias en Biosistemática y Manejo de Recursos Naturales y Agrícolas, Centro Universitario de Ciencias Biológicas y Agropecuarias (CUCBA), Universidad de Guadalajara,
Zapopan 45200, Jalisco, Mexico; sharixrubio@gmail.com

[4] Laboratorio Nacional de Identificación y Caracterización Vegetal (LaniVeg), Departamento de Botánica y Zoología, Centro Universitario de Ciencias Biológicas y Agropecuarias (CUCBA), Universidad de
Guadalajara, Zapopan 45200, Jalisco, Mexico; pilar.zamora@academicos.udg.mx (M.d.P.Z.-T.);
ofelia.vargas@academicos.udg.mx (O.V.-P.)

[5] Laboratorio de Ecología Marina, Centro Universitario de la Costa (CUCosta), Universidad de Guadalajara,
Puerto Vallarta 48280, Jalisco, Mexico; alma.rtroncoso@academicos.udg.mx

* Correspondence: fabian.rzaragoza@academicos.udg.mx

† These authors contributed equally to this work.

Supplementary material

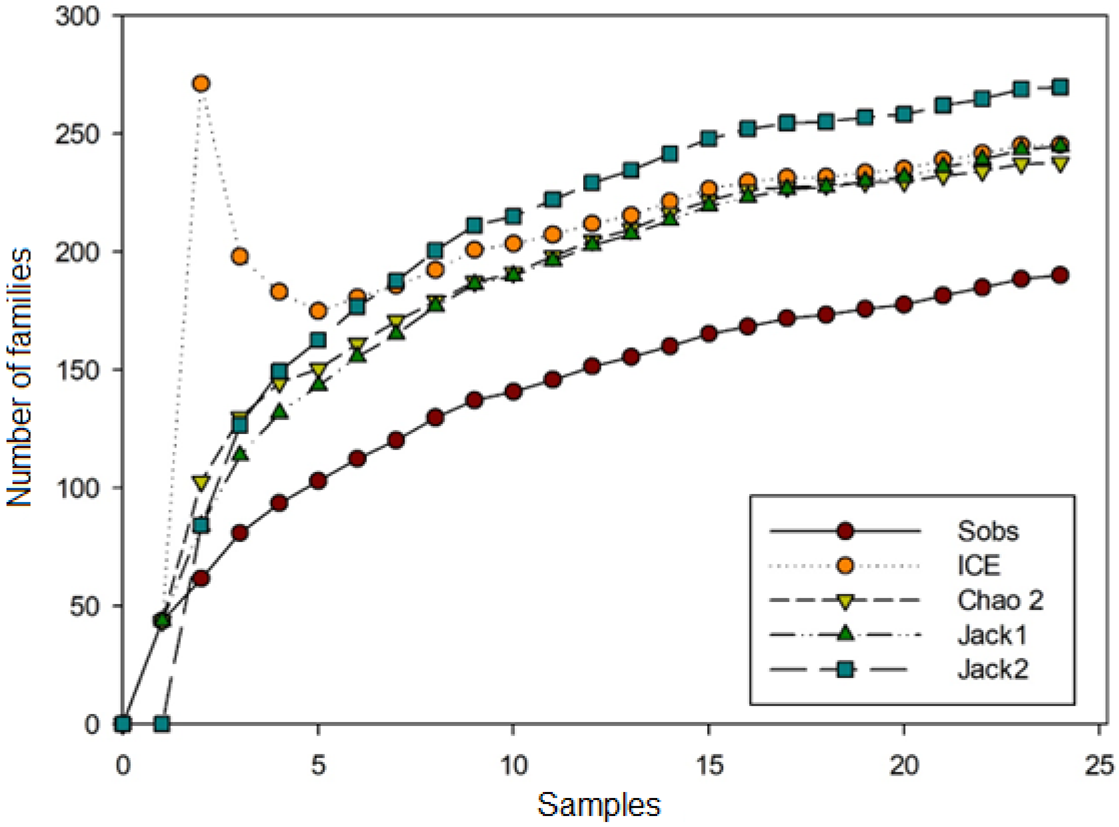

Figure S1. Rarefaction curves based on samples from the entire sampling effort (all sites and times studied). The figure compares the observed (Sobs) and expected richness of the non-parametric estimators (ICE, Chao 2, Jackknife 1, Jackknife 2) of the bacterial families associated with the sea urchin *T. roseus*.

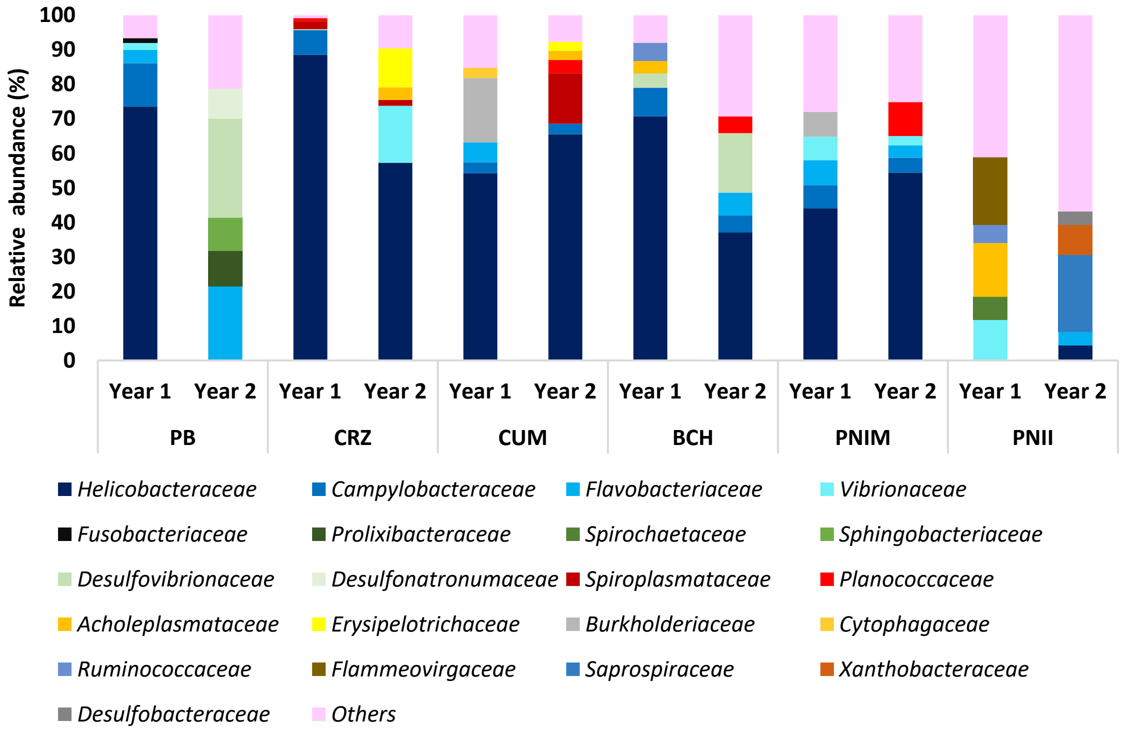

Figure S2. Average relative abundance of the ten most abundant bacterial families of the sea urchin *T. roseus* at a spatio-temporal level (site per year) Codes: PNII: Isla Isabel National Park; PNIM: Islas Marietas National Park; BCH: Islas e islotes de Bahía Chamela Sanctuary; CUM: Bahía Cuastecomates-Punta Melaque; CRZ: Carrizales; PB: Punto B.

Table S1. Environmental variables were used as predictors to evaluate the relationship with the bacterial assemblage of *T. roseus* at the spatial and spatio-temporal models. We included all variables recorded in the study, such as variables with multicollinearity, variables considered for the canonical redundancy analysis (RDA,) and variables selected in the final RDA models. Codes: SST: Sea surface temperature; Salt: Salinity; DO: Dissolved oxygen; CEL: Light extinction coefficient; Clα: Chlorophyll-α; ColiTotal: Total coliforms; ColiFec; Fecal coliforms; Lat: Latitude; Lon: Longitude; Prof: Depth; TIC: Topographic complexity index; CCV: Live coral cover; CH: hydrocoral cover; CHZ: Hydrozoan cover; COct: Octocoral cover; CEsp: Sponge cover; CMacro: Macroalgae Cover; CTurf: Coverage of filamentous algae (grass); CACC: Cover of crusty coralline algae; POOP; Articulated calcareous algae cover; VOC: Vagile organism coverage; COS: Coverage of sessile organisms; CSA: Sandy substrate cover; CEsc: Debris Cover; RSE: Coverage of rocky substrate; Others: Coverage of others; CCMR: Coral cover with recent death; CBC: Coral bleaching coverage.

	Spatial RDA model		
Environmental variables	[illegible]inated environmental vari[illegible] by multicollinearity	[illegible]t environmental variable[illegible] the RDA model	[illegible]cted environmental variables i[illegible] final RDA Model
TSM	TSM	CEL	Prof
Salt	Salt	ColiFec	CCV
OD	OD	PO_4	CEsp
CEL	Clα	Lat	CSA
Clα	ColiTotal	Prof	
ColiTotal	ColiFec	CCV	
ColiFec	NO_3+NO_2	CEsp	
NO_3+NO_2	NH_4	CACC	
NH_4	SiO_2	CACA	
PO_4	Lon	CSA	
SiO_2	ICT	CEsc	
Lat	CH		
Lon	CHZ		
Prof	COct		
ICT	CMacro		
CCV	CTurf		
CH	CACA		
CHZ	COV		
COct	COS		
CEsp	CSR		
CMacro	Others		
CTurf	CCMR		
CACC	CBC		
CACA			
COV			
COS		**Spatio-temporal RDA model**	

CSA	inated environmental vari by multicollinearity	it environmental variabl the RDA model	ected environmental variables i final RDA Model
CEsc	ColiTotal	TSM	TSM
CSR	NO_3+NO_2	Salt	Sal
Others	SiO_2	OD	OD
CCMR	Lat	CEL	CEL
CBC	Lon	Clα	ColiFec
	ICT	ColiFec	CEsp
	CH	NH_4	CMacro
	CHZ	PO_4	CSA
	CACA	Prof	
	COV	CCV	
	COS	COct	
	Others	CEsp	
	CCMR	CMacro	
	CBC	CTurf	
		CACC	
		CSA	
		CEsc	
		CSR	

Notes: 1) For the spatial RDA models, only environmental variables that presented significant differences between study sites were used; 2) Environmental variables that were multicollinear (Pearson correlation ≥ 0.75) were removed from the RDA models; 3) A forward-selection procedure determined the selected environmental variables in the final RDA models.

Table S2. Results of the three-way PERMANOVA with crossed factors for the alpha (N, ^{0}D, ^{1}D, ^{2}D, 21D) and beta (family composition and abundance) diversity of the bacterial assemblage associated with the sea urchin *T. roseus*. Codes: C.V.% = Coefficient of explained variation in percentage. Values in bold correspond to significant differences ($P \leq 0.05$).

Source of variation	Pseudo-*F*	*P*-value	C.V.%
Alpha diversity			
Site	2.2408	0.1311	32.2
Season	0.3089	0.7644	0.0
Year	1.3691	0.2767	10.1
Site*Season	0.4200	0.9144	0.0
Site*Season	0.2318	0.9902	0.0
Season*Year	0.5434	0.6259	0.0
Residuals			57.7
Beta diversity			
Site	1.9401	0.0691	15.7
Season	1.5836	0.1999	7.1
Year	2.7850	0.0605	12.5
Site*Season	1.1568	0.3345	9.0
Site*Season	1.4787	0.1437	15.8
Season*Year	1.3302	0.2825	7.6
Residuals			32.3

Table S3. Total observed and expected richness of bacterial families from the non-parametric estimators (ICE, Chao 2, Jackknife 1, and Jackknife 2) with their respective percentages of representativeness according to all the sites and times studied. The average of the expected wealth and percentage of representativeness of the non-parametric estimators is included in the table.

Estimator / Average	Observed richness / Expected	Representativeness %
Observed richness	190	–
ICE	245.3	77.5
Chao 2	244.6	77.7
Jackknife 1	269.7	70.5
Jackknife 2	245.3	77.5
Average	296.9	76.4

Table S4. *A posteriori* tests of the Site factor of the PERMANOVA models of the alpha and beta diversity of the bacterial assemblage associated with the sea urchin *T. roseus* and the environmental variables. Values in bold correspond to significant differences (P ≤ 0.05). Codes: MC: Monte Carlo tests; PNII: Isla Isabel National Park; PNIM: Islas Marietas National Park; BCH: Islas e islotes de Bahía Chamela Sanctuary; CUM: Bahía Cuastecomates-Punta Melaque; CRZ: Carrizales; PB: Punto B.

Groups	Alpha diversity	Beta diversity	Environmental variables
	P-value	*P*-value	*P*-value (MC)
PNII, PNIM	0.0881	**0.0268**	0.2927
PNII, BCH	**0.0302**	**0.0275**	0.2851
PNII, CUM	**0.0348**	**0.0292**	0.3375
PNII, CRZ	**0.0319**	**0.0284**	0.1957
PNII, PB	**0.0349**	0.0590	0.3643
PNIM, BCH	0.4299	0.3488	0.4321
PNIM, CUM	0.3959	0.9063	0.3836
PNIM, CRZ	0.3930	0.5903	0.2864
PNIM, PB	0.3093	0.4011	0.4635
BCH, CUM	0.8498	0.6364	0.4870
BCH, CRZ	0.5414	0.8164	0.2834
BCH, PB	0.7695	0.5719	0.4582
CUM, CRZ	0.6870	0.9003	0.2853
CUM, PB	0.6764	0.5295	0.3298
CRZ, PB	0.6391	0.5272	0.2794

Table S5. Results obtained from the similarity percentage analysis (SIMPER) at a cut-off of 65% of the cumulative contribution to the average dissimilarity. Comparisons of variables (bacterial families) are shown between paired groups of years (2017-2018 and 2018-2019) and sites (PNII: Isla Isabel National Park; PNIM: Islas Marietas National Park; BCH: Islas e islotes de Bahía Chamela Sanctuary; CUM: Bahía Cuastecomates-Punta Melaque; CRZ: Carrizales; PB: Punto B). Codes: Av.Sq.Dist.: Average square distance; Sq.Dist./SD: Square distance/Standard deviation; Contrib%; Percentage of contribution; Cum.%: Percentage of cumulate contribution.

Families	Average value	Average value	Av.Sq.Dist	Sq.Dist./SD	Contrib%	Cum.%
ps 2017-2018 and 2018-2019						
age square distance = 96.88						
	Group 2017-2018	Group 2018-201				
obacteraceae	6.57	4.97	19	0.89	19.56	19.56
lfovibrionaceae	0.604	2.57	11.2	0.64	11.55	31.11
holderiaceae	1.11	1.05	8.54	0.61	8.82	39.92
bacteriaceae	1.37	1.83	4.4	0.57	4.54	44.46
plasmataceae	0.557	1.67	4.16	0.9	4.3	48.76
lfonatronaceae	8.88E-02	1.15	3.98	0.55	4.11	52.86
ylobacteraceae	2.15	1.32	3.28	0.94	3.39	56.25
leplasmataceae	1.45	0.603	3.15	0.65	3.25	59.5
onaceae	1.54	1.79	2.84	0.8	2.93	62.44
xellaceae	8.26E-02	0.774	2.55	0.34	2.63	65.07
ps PNII and PNIM						
age square distance = 123.62						
	Group PNII	Group PNIM				
obacteraceae	1.15	7.18	37.3	3.14	30.18	30.18
holderiaceae	1.26	1.8	13	0.7	10.48	40.66
meovirgaceae	1.57	0.285	6.74	0.56	5.45	46.11
xellaceae	1.65	0.181	6.7	0.57	5.42	51.53

ospiraceae	1.23	2.93E-02	5.96	0.56	4.82	56.35
leplasmataceae	2.11	1.16	4.56	1.17	3.69	60.04
oylobacteraceae	0.81	2.27	4.19	1.01	3.39	63.43
chaetaceae	1.58	0.118	3.69	0.9	2.98	66.41

ps PNII and BCH

age square distance = 136.46

	Group PNII	Group BCH				
obacteraceae	1.15	6.95	36.8	1.83	26.95	26.95
meovirgaceae	1.57	6.78E-02	7.32	0.57	5.36	32.31
leplasmataceae	2.11	1.04	6.4	0.94	4.69	37
lfovibrionaceae	0.938	2.42	6.28	0.7	4.6	41.6
xellaceae	1.65	0.553	6.04	0.57	4.43	46.03
ospiraceae	1.23	8.46E-02	5.85	0.56	4.29	50.32
holderiaceae	1.26	0	5.58	0.56	4.09	54.41
oylobacteraceae	0.81	2.27	4.19	1.15	3.07	57.47
chaetaceae	1.58	5.95E-02	3.86	0.91	2.83	60.3
lospiraceae	1.23	1.22	3.86	0.79	2.83	63.13
onaceae	2.8	1.24	3.55	0.87	2.6	65.73

ps PNIM and BCH

age square distance = 62.09

	Group PNIM	Group BCH				
holderiaceae	1.8	0	11.9	0.56	19.19	19.19
lfovibrionaceae	0.353	2.42	8.49	0.75	13.67	32.87
lospiraceae	0.329	1.22	3.96	0.57	6.38	39.25
obacteraceae	7.18	6.95	3.29	0.79	5.3	44.55
plasmataceae	1.77	0.989	3.02	0.93	4.86	49.42
obacteriaceae	1.82	1.44	2.49	0.9	4.01	53.43
oylobacteraceae	2.27	2.27	2.29	0.8	3.69	57.12

ılfonatronaceae	2.36E-02	1.17	2.17	0.81	3.49	60.62
ıgobacteriaceae	1.13	0	1.71	1.18	2.75	63.37
ɔcoccaceae	0.674	0.926	1.7	0.7	2.73	66.1

ps PNII and CUM

ıage square distance = 138.16

	Group PNII	Group CUM				
·obacteraceae	1.15	7.42	41.7	2.17	30.21	30.21
holderiaceae	1.26	1.54	10.8	0.73	7.84	38.05
meovirgaceae	1.57	9.92E-02	7.24	0.57	5.24	43.29
ıxellaceae	1.65	1.69E-02	7.18	0.58	5.2	48.49
ɔspiraceae	1.23	0	6.03	0.56	4.37	52.86
leplasmataceae	2.11	0.907	5.43	0.97	3.93	56.78
plasmataceae	0.678	1.79	5.03	0.95	3.64	60.43
onaceae	2.8	1.07	4.63	0.91	3.35	63.78
chaetaceae	1.58	0.405	3.23	0.84	2.34	66.12

ps PNIM and CUM

ıage square distance = 51.33

	Group PNIM	Group CUM				
holderiaceae	1.8	1.54	15.5	0.74	30.16	30.16
plasmataceae	1.77	1.79	4.47	1.06	8.71	38.88
lfovibrionaceae	0.353	1.17	3.38	0.59	6.59	45.47
·obacteraceae	7.18	7.42	2.57	0.95	5.01	50.48
ɔylobacteraceae	2.27	1.61	2.53	0.74	4.93	55.41
ɔbacteriaceae	1.82	1.38	2.15	0.89	4.2	59.61
onaceae	1.86	1.07	1.89	0.87	3.69	63.3
ɔcaceae	0.974	0	1.57	0.95	3.06	66.35

ps BCH and CUM

ıage square distance = 60.95

	Group BCH	Group CUM				
holderiaceae	0	1.54	9.14	0.56	14.99	14.99
lfovibrionaceae	2.42	1.17	7.92	0.78	13	27.99
plasmataceae	0.989	1.79	5.07	0.97	8.32	36.31
obacteraceae	6.95	7.42	4.98	0.77	8.17	44.47
lospiraceae	1.22	0.519	3.71	0.59	6.09	50.56
ylobacteraceae	2.27	1.61	2.53	0.79	4.15	54.71
bacteriaceae	1.44	1.38	2.45	0.9	4.02	58.74
coccaceae	0.926	0.918	2.32	0.82	3.8	62.54
leplasmataceae	1.04	0.907	2	0.78	3.27	65.81

ps PNII and CRZ

age square distance = 145.12

	Group PNII	Group CRZ				
obacteraceae	1.15	6.78	41.2	1.47	28.41	28.41
lfovibrionaceae	0.938	2.01	8.96	0.6	6.18	34.58
xellaceae	1.65	1.75E-02	7.18	0.58	4.95	39.53
holderiaceae	1.26	1.72	7.15	0.91	4.92	44.45
meovirgaceae	1.57	0.365	6.79	0.57	4.68	49.13
leplasmataceae	2.11	0.735	6.16	0.93	4.25	53.38
spiraceae	1.23	1.79E-02	5.99	0.56	4.13	57.51
lfonatronaceae	2.56E-02	1.15	5.22	0.56	3.6	61.1
onaceae	2.8	1.61	4.73	0.97	3.26	64.36
chaetaceae	1.58	0.159	3.58	0.89	2.47	66.83

ps PNIM and CRZ

age square distance = 66.16

	Group PNIM	Group CRZ				
holderiaceae	1.8	1.72	11.6	0.77	17.56	17.56
lfovibrionaceae	0.353	2.01	10.7	0.59	16.15	33.7
obacteraceae	7.18	6.78	9.83	0.73	14.85	48.56

lfonatronaceae	2.36E-02	1.15	5.22	0.56	7.89	56.45
plasmataceae	1.77	1.27	3.81	0.95	5.76	62.21
onaceae	1.86	1.61	3.02	1.06	4.56	66.78

ps BCH and CRZ

age square distance = 68.92

	Group BCH	Group CRZ				
obacteraceae	6.95	6.78	11.9	0.77	17.31	17.31
lfovibrionaceae	2.42	2.01	11.7	0.84	17.04	34.35
holderiaceae	0	1.72	5.89	0.97	8.54	42.89
lfonatronaceae	1.17	1.15	4.81	0.79	6.98	49.87
lospiraceae	1.22	0.186	4.21	0.57	6.1	55.98
plasmataceae	0.989	1.27	3.59	0.77	5.21	61.19
onaceae	1.24	1.61	3.1	0.78	4.49	65.69

ps CUM and CRZ

age square distance = 66.57

	Group CUM	Group CRZ				
obacteraceae	7.42	6.78	11.6	0.69	17.41	17.41
lfovibrionaceae	1.17	2.01	10.8	0.67	16.22	33.63
holderiaceae	1.54	1.72	9.73	0.83	14.61	48.24
plasmataceae	1.79	1.27	5.85	0.94	8.79	57.03
lfonatronaceae	0.239	1.15	4.92	0.57	7.4	64.43
onaceae	1.07	1.61	3.76	0.75	5.65	70.08

ps PNII and PB

age square distance = 139.39

	Group PNII	Group PB				
obacteraceae	1.15	5.14	25.7	0.94	18.44	18.44
lfovibrionaceae	0.938	2.62	12.3	0.57	8.8	27.24
bacteriaceae	1.84	2.5	7.56	1.03	5.42	32.66

leplasmataceae	2.11	0.219	7.35	0.95	5.27	37.94
meovirgaceae	1.57	0.134	7.16	0.57	5.14	43.08
xellaceae	1.65	0.147	6.83	0.57	4.9	47.98
spiraceae	1.23	0.124	5.79	0.56	4.15	52.13
holderiaceae	1.26	0.155	5.28	0.56	3.79	55.92
lfonatronaceae	2.56E-02	1.1	4.8	0.56	3.44	59.36
onaceae	2.8	1.41	4.22	0.82	3.03	62.39
ylobacteraceae	0.81	1.66	3.9	0.75	2.8	65.19

ps PNIM and PB

age square distance = 87.94

	Group PNIM	Group PB				
lfovibrionaceae	0.353	2.62	14.7	0.58	16.71	16.71
obacteraceae	7.18	5.14	14	1	15.95	32.66
holderiaceae	1.8	0.155	11.5	0.56	13.02	45.68
bacteriaceae	1.82	2.5	8.42	0.92	9.57	55.26
lfonatronaceae	2.36E-02	1.1	4.8	0.56	5.46	60.71
xibacteraceae	0.603	1.54	4.23	0.7	4.81	65.52

ps BCH and PB

age square distance = 84.14

	Group BCH	Group PB				
obacteraceae	6.95	5.14	15.4	0.93	18.25	18.25
lfovibrionaceae	2.42	2.62	13.2	0.75	15.73	33.98
bacteriaceae	1.44	2.5	9.57	0.84	11.37	45.35
gobacteriaceae	0	1.48	4.9	0.65	5.82	51.17
lfonatronaceae	1.17	1.1	4.5	0.81	5.35	56.52
xibacteraceae	0.803	1.54	4.33	0.72	5.15	61.66
lospiraceae	1.22	0.598	3.81	0.61	4.53	66.2

ps CUM and PB

age square distance = 91.21

	Group CUM	Group PB				
obacteraceae	7.42	5.14	16.6	0.92	18.16	18.16
lfovibrionaceae	1.17	2.62	13.8	0.62	15.15	33.31
bacteriaceae	1.38	2.5	9.3	0.84	10.2	43.5
holderiaceae	1.54	0.155	8.75	0.56	9.6	53.1
plasmataceae	1.79	0.186	5.93	0.94	6.5	59.6
lfonatronaceae	0.239	1.1	4.52	0.57	4.96	64.56
xibacteraceae	0.55	1.54	4.45	0.7	4.88	69.44

ps CRZ and PB

age square distance = 97.44

	Group CRZ	Group PB				
obacteraceae	6.78	5.14	21.2	0.94	21.74	21.74
lfovibrionaceae	2.01	2.62	17.3	0.74	17.77	39.51
bacteriaceae	0.595	2.5	11	0.79	11.3	50.81
lfonatronaceae	1.15	1.1	7.6	0.75	7.79	58.6
holderiaceae	1.72	0.155	5.45	0.96	5.6	64.2
xibacteraceae	2.58E-02	1.54	5.39	0.7	5.53	69.73

Table S6. Results obtained from the similarity percentage analysis (SIMPER) at a cut-off of 65% of the cumulative contribution to the average dissimilarity. Comparisons of environmental variables are shown between paired groups of years (2017-2018 and 2018-2019) and sites (PNII: Isla Isabel National Park; PNIM: Islas Marietas National Park; BCH: Islas e islotes de Bahía Chamela Sanctuary; CUM: Bahía Cuastecomates-Punta Melaque; CRZ: Carrizales; PB: Punto B). Codes: Av.Sq.Dist.: Average square distance; Sq.Dist./SD: Square distance/Standard deviation; Contrib%; Percentage of contribution; Cum.%: Percentage of cumulate contribution.

Families	Average value	verage val	Av.Sq.Dist.	Sq.Dist./SD	Contrib%	Cum.%
ps 2017-2018 and 2018-2019						
ıge square distance = 27.96						
	Group 2017-20	bup 2018-2				
rophyll α	-0.849	0.849	3.25	1.47	11.61	11.61
ıonium	0.002	-0.002	2.25	0.65	8.05	19.66
ılved oxygen	-0.689	0.689	2.19	1.29	7.85	27.51
te and nitrite	-0.261	0.261	2.14	1.18	7.67	35.18
erature	0.156	-0.156	2.07	0.9	7.41	42.59
ose calcareous algae	0.361	-0.361	2.01	1.31	7.2	49.79
oalgae	0.27	-0.27	1.87	0.6	6.68	56.47
ity	-0.317	0.317	1.79	0.41	6.42	62.89
ıte	0.073	-0.073	1.58	0.7	5.65	68.54
ps PNII and PNIM						
ıge square distance = 34.45						
	Group PNII	Group PNI				
ity	-1.5	0.483	6.66	0.72	19.32	19.32
ge	1.96	-0.308	5.22	2.86	15.17	34.49
ulated calcareous algae	-0.738	1.13	4.97	0.77	14.43	48.92
	-0.962	0.977	4.1	1.28	11.91	60.83
erature	0.663	-1.11	3.14	21.84	9.12	69.94

ps PNII and BCH

ıge square distance = 54.07

	Group PNII	Group BC				
phate	1.42	-0.915	8.53	0.74	15.78	15.78
ge	1.96	-0.591	6.9	1.52	12.76	28.54
ity	-1.5	0.359	5.87	0.72	10.87	39.4
h	1.29	-0.942	4.97	1.3	9.2	48.6
[illegible]	-0.531	1.35	4.6	0.84	8.51	57.12
te and nitrite	0.729	-1.01	4.37	0.76	8.08	65.2

ps PNIM and BCH

ıge square distance = 39.55

	Group PNIM	Group BC				
:ose calcareous algae	-0.146	0.761	4.61	0.92	11.65	11.65
[illegible]	-0.724	1.35	4.57	1.54	11.55	23.19
h	1.17	-0.942	4.48	1.28	11.32	34.52
ıonium	-0.223	0.936	3.91	0.74	9.89	44.41
ulated calcareous algae	1.13	-0.324	3.64	0.72	9.2	53.61
[illegible]	0.977	0.562	3.61	1.66	9.13	62.74
›erature	-1.11	3.32E-02	2.78	0.71	7.02	69.76

ps PNII and CUM

ıge square distance = 41.79

	Group PNII	Group CU				
ge	1.96	-0.609	6.95	1.6	16.64	16.64
h	1.29	-1.18	6.11	Undefined!	14.63	31.27
ity	-1.5	0.314	5.78	0.71	13.83	45.1
: extinction coefficient	-0.974	1.01	5.35	0.8	12.79	57.9
phate	1.42	-0.279	4.32	0.75	10.33	68.22

ps PNIM and CUM

age square distance = 29.46

	Group PNIM	Group CU				
extinction coefficient	-0.927	1.01	5.82	0.74	19.77	19.77
h	1.17	-1.18	5.56	1.32	18.89	38.66
te and nitrite	-0.419	0.186	2.75	1.04	9.33	47.99
ulated calcareous algae	1.13	-0.192	2.61	0.75	8.87	56.86
	0.977	-0.481	2.31	1.32	7.83	64.69
erature	-1.11	-0.297	2.09	0.76	7.08	71.77

ps BCH and CUM

age square distance = 20.75

	Group BCH	Group CU				
oalgae	-0.676	1.01	4.83	0.72	23.27	23.27
onium	0.936	-0.359	3.73	0.71	17.97	41.23
	1.35	4.68E-02	3.54	0.71	17.04	58.27
	0.562	-0.481	3.13	0.74	15.1	73.37

ps PNII and CRZ

age square distance = 45.15

	Group PNII	Group CR				
coral	-0.851	2.02	8.25	8.83	18.26	18.26
ge	1.96	-0.702	7.34	1.93	16.25	34.51
entous algae	1	-1.63	6.94	5.65	15.38	49.89
ity	-1.5	0.317	5.63	0.72	12.46	62.35
phate	1.42	-0.398	4.96	0.75	10.99	73.34

ps PNIM and CRZ

age square distance = 30.75

	Group PNIM	Group CR				
ulated calcareous algae	1.13	-0.853	5.33	0.8	17.33	17.33
coral	-0.281	2.02	5.29	16.56	17.2	34.52

ıentous algae	0.363	-1.63	3.96	11.4	12.87	47.4
	0.977	-0.737	3.16	1.4	10.27	57.66
:ose calcareous algae	-0.146	-1.29	3.02	0.71	9.83	67.49

ps BCH and CRZ

ıge square distance = 29.57

	Group BCH	Group CR
:ose calcareous algae		
		[illegible]
ıonium		[illegible]
coral	-4.8	
		[illegible]

ps CUM and CRZ

ıge square distance = 22.35

	Group CUM	Group CR
oalgae		[illegible]
coral	-6.8	
:ose calcareous algae		
te and nitrite		[illegible]
: extinction coefficient		[illegible]

ps PNII and PB

ıge square distance = 46.38

	Group PNII	Group PB
. coliforms	[illegible]	
ity		2.5
phate		-8.0
ɔerature		
ulated calcareous algae	[illegible]	
ıte	-1.7	

ge

ıonium

ps PNIM and PB

ıge square distance = 44.48

	Group PNIM	Group PE
›erature		
. coliforms		
›lved oxygen		
ıte		
te and nitrite		
ulated calcareous algae		

ps BCH and PB

ıge square distance = 39.03

	Group BCH	Group PE
ıte		
ıonium		
. coliforms		
te and nitrite		

ps CUM and PB

ıge square distance = 32.70

	Group CUM	Group PE
. coliforms		
ıte		
›oalgae		
ıonium		
ıentous algae		

ps CRZ and PB

ıge square distance = 48.78

	Group CRZ	Group PE
coral		[illegible]
ıentous algae		
ıte	-5.8	
. coliforms	[illegible]	
ulated calcareous algae	[illegible]	
ıonium	[illegible]	

Table S7. Results obtained from the similarity percentage analysis (SIMPER) at a cut-off of 65% of the cumulative contribution of the bacterial families to the average dissimilarity. Comparisons of bacterial families were made between paired groups of years (2017-2018 and 2018-2019) and sites (PNII: Isla Isabel National Park; PNIM: Islas Marietas National Park; BCH: Islas e islotes de Bahía Chamela Sanctuary; CUM: Bahía Cuastecomates-Punta Melaque; CRZ: Carrizales; PB: Punto B). Codes: Av.Sq.Dist.: Average square distance; Sq.Dist./SD: Square distance/Standard deviation; Contrib%; Percentage of contribution; Cum.%: Percentage of cumulate contribution.

Families	Average value	Average value	Av.Sq.Dist	Sq.Dist./SD	Contrib%	Cum.%
ps 2017-2018 and 2018-2019						
age square distance = 64.46						
	Group 2017-2018	Group 2018-201				
obacteraceae	6.85	5.57	8.72	0.55	13.53	13.53
holderiaceae	1.19	0.887	6.14	0.86	9.53	23.06
lfovibrionaceae	0.73	2.31	5.02	0.54	7.79	30.85
plasmataceae	0.535	2.09	4.1	0.74	6.37	37.22
ospiraceae	0.009	0.912	3.76	0.41	5.84	43.06
onaceae	1.61	1.95	3.26	0.56	5.05	48.11
leplasmataceae	1.47	0.857	3.2	0.68	4.96	53.07
obacteriaceae	1.58	2.08	3.16	1.09	4.91	57.97
meovirgaceae	0.858	0.258	2.67	0.42	4.14	62.12
oylobacteraceae	2.49	1.33	2.29	0.64	3.55	65.67
ps PNII and PNIM						
age square distance = 94.85						
	Group PNII	Group PNIM				
obacteraceae	1.41	7.01	31.4	6.45	33.13	33.13
ospiraceae	2.37	5.57E-02	10.9	0.71	11.53	44.65
meovirgaceae	2.45	0.328	8.02	0.71	8.45	53.11
holderiaceae	0.708	1.42	3.92	1.05	4.13	57.24
leplasmataceae	2.19	1.25	3.7	0.83	3.91	61.15

hobacteraceae	1.56	0.122	3.65	0.71	3.85	65
chaetaceae	1.73	0.227	3.09	0.79	3.26	68.26

ps PNII and BCH

age square distance = 115.89

	Group PNII	Group BCH				
obacteraceae	1.41	7.25	37.5	1.23	32.36	32.36
spiraceae	2.37	0.122	10.1	0.71	8.69	41.04
meovirgaceae	2.45	9.39E-02	9.86	0.71	8.51	49.55
lfovibrionaceae	1.04	3.09	5.09	0.93	4.39	53.94
hobacteraceae	1.56	0	4.34	0.71	3.74	57.68
chaetaceae	1.73	8.23E-02	3.58	0.82	3.09	60.77
onaceae	2.68	1.24	3.41	0.72	2.94	63.71
ylobacteraceae	0.892	2.54	2.79	2.45	2.41	66.12

ps PNIM and BCH

age square distance = 44.37

	Group PNIM	Group BCH				
lfovibrionaceae	0.693	3.09	6	1.77	13.53	13.53
holderiaceae	1.42	0	3.65	0.71	8.22	21.75
bacteriaceae	2.3	1.46	3	0.83	6.76	28.51
gobacteriaceae	1.58	0	2.55	2.44	5.74	34.25
obacteraceae	7.01	7.25	2.4	2.27	5.41	39.66
caceae	1.64	0.163	2.31	1.51	5.2	44.86
lfonatronumaceae	0.05	1.45	2.22	1.11	5	49.86
midiaceae	1.26	0	1.61	2.48	3.64	53.5
nococcaceae	0.63	1.31	1.57	0.77	3.54	57.04
onaceae	2.12	1.24	1.54	0.71	3.48	60.52
arinaceae	1.17	0	1.39	3.22	3.12	63.64
domonadaceae	0.13	0.987	1.32	0.71	2.97	66.62

ps PNII and CUM

ge square distance = 122.66

	Group PNII	Group CUM				
obacteraceae	1.41	7.73	40.1	7.15	32.66	32.66
spiraceae	2.37	0	11.2	0.71	9.12	41.79
holderiaceae	0.708	2.22	9.4	0.82	7.67	49.46
meovirgaceae	2.45	0.142	8.71	0.73	7.1	56.56
leplasmataceae	2.19	1.21	5.74	0.95	4.68	61.24
hobacteraceae	1.56	0.08	3.91	0.71	3.19	64.43
onaceae	2.68	0.867	3.47	1.52	2.83	67.26

ps PNIM and CUM

ge square distance = 25.05

	Group PNIM	Group CUM				
caceae	1.64	0	2.72	2.9	10.87	10.87
ylococcaceae	0.917	0.764	1.99	3.3	7.95	18.82
midiaceae	1.26	0	1.61	2.48	6.45	25.27
naceae	2.12	0.867	1.59	2.69	6.36	31.62
bacteriaceae	2.3	1.29	1.51	0.75	6.04	37.66
arinaceae	1.17	0	1.39	3.22	5.53	43.19
holderiaceae	1.42	2.22	1.31	0.71	5.25	48.44
pelotrichaceae	1.01	0.798	1.17	1.88	4.65	53.09
gobacteriaceae	1.58	0.519	1.14	2.81	4.56	57.65
hagaceae	0.468	0.862	0.975	0.97	3.89	61.54
xibacteraceae	1.06	0.588	0.591	0.73	2.36	63.9
plasmataceae	1.97	1.91	0.57	4.69	2.28	66.17

ps BCH and CUM

ge square distance = 47.57

	Group BCH	Group CUM				
holderiaceae	0	2.22	9.33	0.71	19.62	19.62

lfovibrionaceae	3.09	0.585	6.83	1.29	14.37	33.99
obacteriaceae	1.46	1.29	4.94	4.69	10.38	44.37
obacteraceae	7.25	7.73	2.56	1.23	5.37	49.74
lfonatronumaceae	1.45	0.096	2.03	1.17	4.27	54.01
xibacteraceae	0.812	0.588	2.01	2.26	4.22	58.24
leplasmataceae	0.949	1.21	1.96	1.96	4.12	62.36
plasmataceae	1.21	1.91	1.6	0.76	3.36	65.71

ps PNII and CRZ

age square distance = 139.59

	Group PNII	Group CRZ				
obacteraceae	1.41	8.49	52.7	1.65	37.73	37.73
ospiraceae	2.37	0.035	10.9	0.71	7.78	45.51
meovirgaceae	2.45	0.131	9.84	0.71	7.05	52.55
onaceae	2.68	2.12	7.6	1.78	5.44	58
leplasmataceae	2.19	1.16	7.36	1.01	5.27	63.27
hobacteraceae	1.56	0	4.34	0.71	3.11	66.38

ps PNIM and CRZ

age square distance = 43.55

	Group PNIM	Group CRZ				
holderiaceae	1.42	1.68	8.8	4.07	20.21	20.21
onaceae	2.12	2.12	5.86	3331.34	13.44	33.66
obacteriaceae	2.3	0.583	3.91	0.82	8.98	42.64
obacteraceae	7.01	8.49	3.86	0.71	8.86	51.5
ocaceae	1.64	0.035	2.62	2.43	6.02	57.53
gobacteriaceae	1.58	0.182	2.12	1.29	4.88	62.41
plasmataceae	1.97	1.42	1.88	0.96	4.32	66.73

ps BCH and CRZ

age square distance = 37.58

	Group BCH	Group CRZ

lfovibrionaceae	3.09	0.797	5.9	1.14	15.7	15.7
holderiaceae	0	1.68	5.64	0.71	15	30.7
onaceae	1.24	2.12	3.17	0.82	8.43	39.13
leplasmataceae	0.949	1.16	2.93	2.91	7.8	46.93
nococcaceae	1.31	0.172	2.08	0.73	5.54	52.47
ococcaceae	1.1	0.722	1.91	1.35	5.08	57.55
domonadaceae	0.987	0	1.64	0.72	4.35	61.9
obacteraceae	7.25	8.49	1.59	1.89	4.23	66.13

ps CUM and CRZ

age square distance = 44.50

	Group CUM	Group CRZ				
holderiaceae	2.22	1.68	14.5	2.51	32.69	32.69
onaceae	0.867	2.12	6.64	0.83	14.92	47.61
plasmataceae	1.91	1.42	4.28	1.52	9.62	57.23
obacteriaceae	1.29	0.583	3.32	0.99	7.47	64.7
obacteraceae	7.73	8.49	2.22	0.81	4.99	69.69

ps PNII and PB

age square distance = 116.91

	Group PNII	Group PB				
obacteraceae	1.41	5.39	30.8	0.71	26.32	26.32
meovirgaceae	2.45	0.2	9.82	0.71	8.4	34.72
ospiraceae	2.37	0.185	9.51	0.71	8.13	42.85
lfovibrionaceae	1.04	2.93	8.89	0.72	7.61	50.46
leplasmataceae	2.19	0.228	6.19	0.73	5.29	55.75
hobacteraceae	1.56	0	4.34	0.71	3.71	59.46
lfonatronumaceae	0.05	1.47	4.04	0.71	3.45	62.91
obacteriaceae	2.03	3.3	3.57	0.71	3.05	65.97

ps PNIM and PB

age square distance = 63.89

	Group PNIM	Group PB				
obacteraceae	7.01	5.39	15.3	0.94	23.9	23.9
lfovibrionaceae	0.693	2.93	8.5	0.72	13.3	37.2
lfonatronumaceae	0.05	1.47	4.05	0.71	6.34	43.54
bacteriaceae	2.3	3.3	4	0.82	6.27	49.81
plasmataceae	1.97	0.248	3.79	0.86	5.93	55.75
holderiaceae	1.42	0.207	3.67	0.72	5.75	61.5
caceae	1.64	0	2.72	2.9	4.26	65.76

ps BCH and PB

ge square distance = 37.14

	Group BCH	Group PB				
obacteraceae	7.25	5.39	7.57	0.71	20.4	20.4
gobacteriaceae	0	1.95	5.15	0.8	13.86	34.26
bacteriaceae	1.46	3.3	3.45	3.02	9.28	43.54
lfovibrionaceae	3.09	2.93	1.9	3.02	5.12	48.67
ylobacteraceae	2.54	2.01	1.71	0.95	4.59	53.26
xibacteraceae	0.812	2.09	1.7	1.68	4.59	57.85
coccaceae	1.1	0.227	1.51	0.71	4.08	61.93
nococcaceae	1.31	0.745	1.43	0.84	3.84	65.77

Groups CUM and PB

Average square distance = 84.98

	Group CUM	Group PB				
obacteraceae	7.73	5.39	18.1	0.77	21.3	21.3
bacteriaceae	1.29	3.3	9.97	0.72	11.74	33.04
lfovibrionaceae	0.585	2.93	9.97	0.71	11.73	44.77
holderiaceae	2.22	0.207	9.37	0.71	11.02	55.79
plasmataceae	1.91	0.248	5.54	0.71	6.52	62.3
xibacteraceae	0.588	2.09	5.08	0.71	5.97	68.28

ps CRZ and PB

ıge square distance = 62.74

	Group CRZ	Group PB				
·obacteraceae	8.49	5.39	14.7	0.74	23.5	23.5
lfovibrionaceae	0.797	2.93	9.25	0.71	14.74	38.25
›bacteriaceae	0.583	3.3	7.98	1.37	12.71	50.96
xibacteraceae	0.051	2.09	5.23	0.87	8.34	59.3
holderiaceae	1.68	0.207	4.33	0.71	6.9	66.2

Table S8. Conditional effects of the environmental variables selected for the canonical redundancy analyzes (RDA) models at the spatial and spatio-temporal levels. Values in bold correspond to significant variables.($P \leq 0.05$).

Variable	Lambda	*F*-value	*P*-value
Spatial model			
Sponge cover	0.50	3.96	**0.004**
Depth	0.15	1.23	0.326
Sand cover	0.14	1.23	0.360
Live coral cover	0.13	1.74	0.354
Spatial-temporal model			
Sponge cover	0.13	4.19	**0.003**
Salinity	0.13	2.37	**0.021**
Dissolved oxygen	0.10	2.22	**0.034**
Fecal coliforms	0.14	2.41	0.078
Phosphates	0.07	2.11	0.120
Macroalgae	0.09	2.00	0.140
Light extinction coefficient	0.05	1.75	0.200
Spatial model α	0.04	1.61	0.225
